# Supplementary material for: Tailor-made nanostructures bridging chaos and order for highly efficient white organic light-emitting diodes
Source: Nat Commun. 2019 Jul 5;10:2972. doi: 10.1038/s41467-019-11032-z (PMC6611821; doi:10.1038/s41467-019-11032-z)
Supplement: Supplementary file 1 — Supplementary Information [file 41467_2019_11032_MOESM1_ESM.pdf]

Supplementary information

Tailor-made nanostructures bridging chaos and order for highly efficient white organic light-emitting diodes

Yungui Li[1], Milan Kovačič[2], Jasper Westphalen[3], Steffen Oswald[4], Zaifei Ma[5], Christian Hänisch[1], Paul-Anton Will[1], Lihui Jiang[1,6], Manuela Junghaehnel[3], Reinhard Scholz[1], Simone Lenk[1] and Sebastian Reineke[1]*

1. *Dresden Integrated Center for Applied Physics and Photonic Materials (IAPP) and Institute for Applied Physics, Nöthnitzer Str. 61, Technische Universität Dresden, Dresden 01062, Germany.*
2. *University of Ljubljana, Faculty of Electrical Engineering, Tržaška 25, 1000 Ljubljana, Slovenia*
3. *Fraunhofer Institute for Organic Electronics, Electron Beam and Plasma Technology FEP, Winterbergstraße 28, 01277 Dresden, Germany*
4. *Institute for Complex Materials, Leibniz IFW Dresden, Helmholtzstraße 20, 01069 Dresden, Germany*
5. *Center for Advanced Low-dimension Materials, State Key Laboratory for Modification of Chemical Fibers and Polymer Materials, Donghua University, Shanghai, 201620，China*
6. *College of Chemistry and Chemical Engineering, Central South University, Changsha, China.*

*To whom all correspondence should be addressed: E-mail: sebastian.reineke@tu-dresden.de

Supplementary Figures:

Supplementary Figure 1. The depth (height) distribution of nanostructures 3
Supplementary Figure 2. Nanostructure uniformity in different positions 3
Supplementary Figure 3. Nanostrucutre reproducibility 4
Supplementary Figure 4. Nanostructure control 5
Supplementary Figure 5. Simulated optical field distribution 6
Supplementary Figure 6. Simulated wavelength dependent enhancement. 7
Supplementary Figure 7. The transmission and reflectance of the sputtered ITO 7
Supplementary Figure 8. OLEDs performance. 8
Supplementary Figure 9. OLEDs angular dependent EL spectra 9
Supplementary Figure 10. Properties of the nanostructures used in OLEDs. 10
Supplementary Figure 11. The EL spectrum fitting for the planar white tandem OLED 11
Supplementary Figure 12. Efficiency of light outcoupling structures 11
Supplementary Figure 13. Experimental aspect ratio 11

Supplementary Tables:

Supplementary Table 1. Nanostructure uniformity: AR based on FWHM 12
Supplementary Table 2. Nanostructure uniformity: AR based on dominant depth 12
Supplementary Table 3. The atomic concentration of PDMS before and after RIE treatment 13
Supplementary Table 4. The RIE treated PDMS with identical XPS spectra 13
Supplementary Table 5. The ratio of $\eta_{\mathrm{SA}} / \eta_{\mathrm{A}}$ for white OLEDs. 13
Supplementary Table 6. Comparison of internal outcoupling structures for white OLEDs. 14
Supplementary Table 7. Parameters for optical analysis of the planar white OLED.. 15
Supplementary Table 8. The fraction of power for each unit of the planar white OLED.. 15

Supplementary Notes:

Supplementary Note 1. Nanostructure fabrication 16
Supplementary Note 2. Optical analysis of loss channels for planar white OLEDs 18
Supplementary Note 3. Efficiency of light outcoupling structures (ELOS). 19
Supplementary Note 4. Experimental aspect ratio 20

1. Supplementary Figures

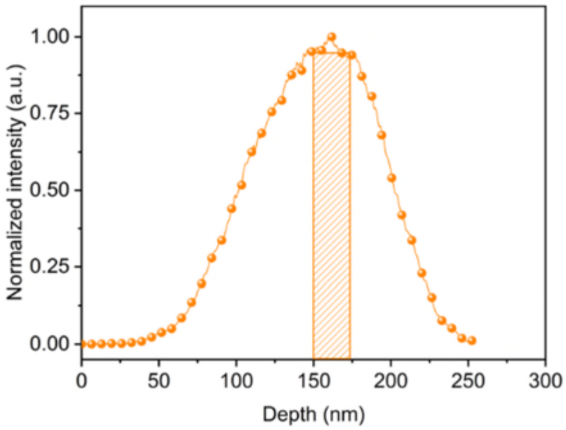

Supplementary Figure 1. The depth (height) distribution of nanostructures. There is a dominant depth among the depth distribution, which is marked with a bar.

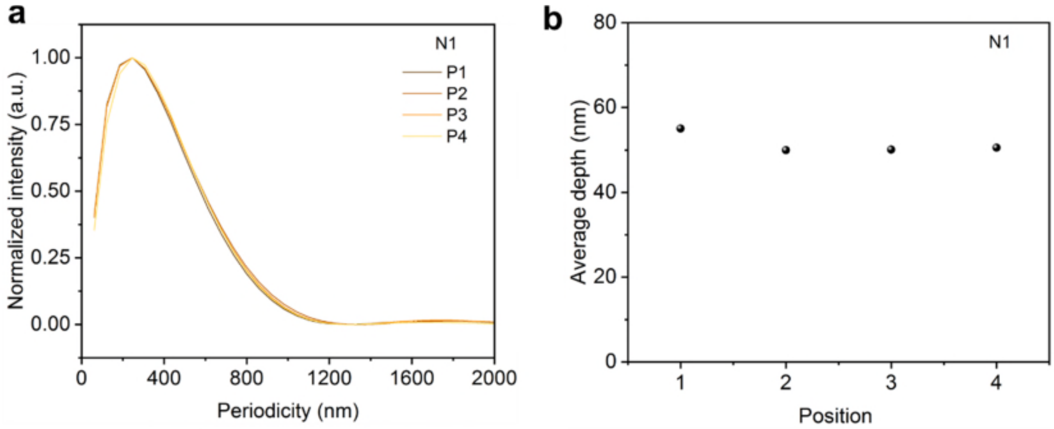

Supplementary Figure 2. Nanostructure uniformity in different positions. Uniformity investigation on sample N1 as a representative nanostructure generated by RIE treatment on PDMS. The structure is investigated with AFM at different local positions (P1 – P4). (a) Periodicity distribution. (b) Average depth. The AFM measurement is done with a resolution of 1024×1024.

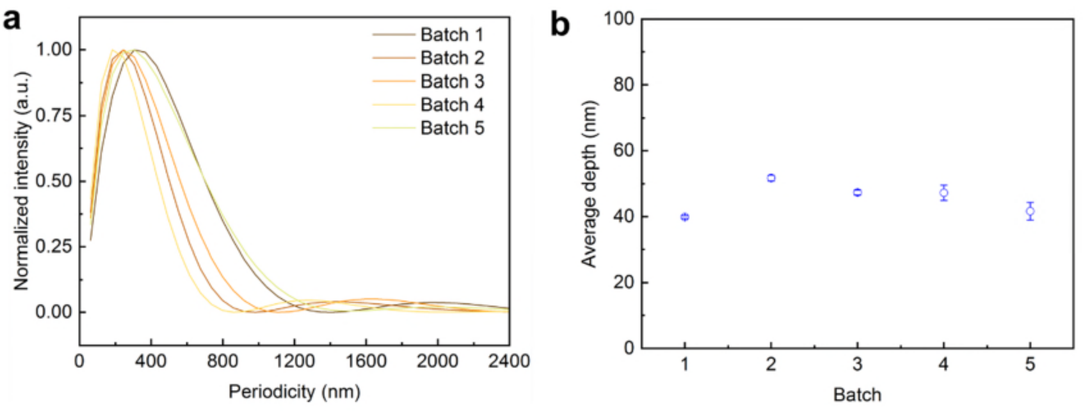

Supplementary Figure 3. Nanostructure reproducibility. Experimental variation of the nanostructures of samples prepared with the same recipe during multiple, consecutive runs (batches 1-5). (a) Periodicity distribution. (b) The average depth. The error bar is the standard deviation from at least three AFM measurements at different positions for each sample. The PDMS preparation recipe: PDMS from Sigma-Aldrich, 1000 rpm, 80°C, 80 min. The RIE recipe: 50 sccm O_2, 50 W, 60 s.

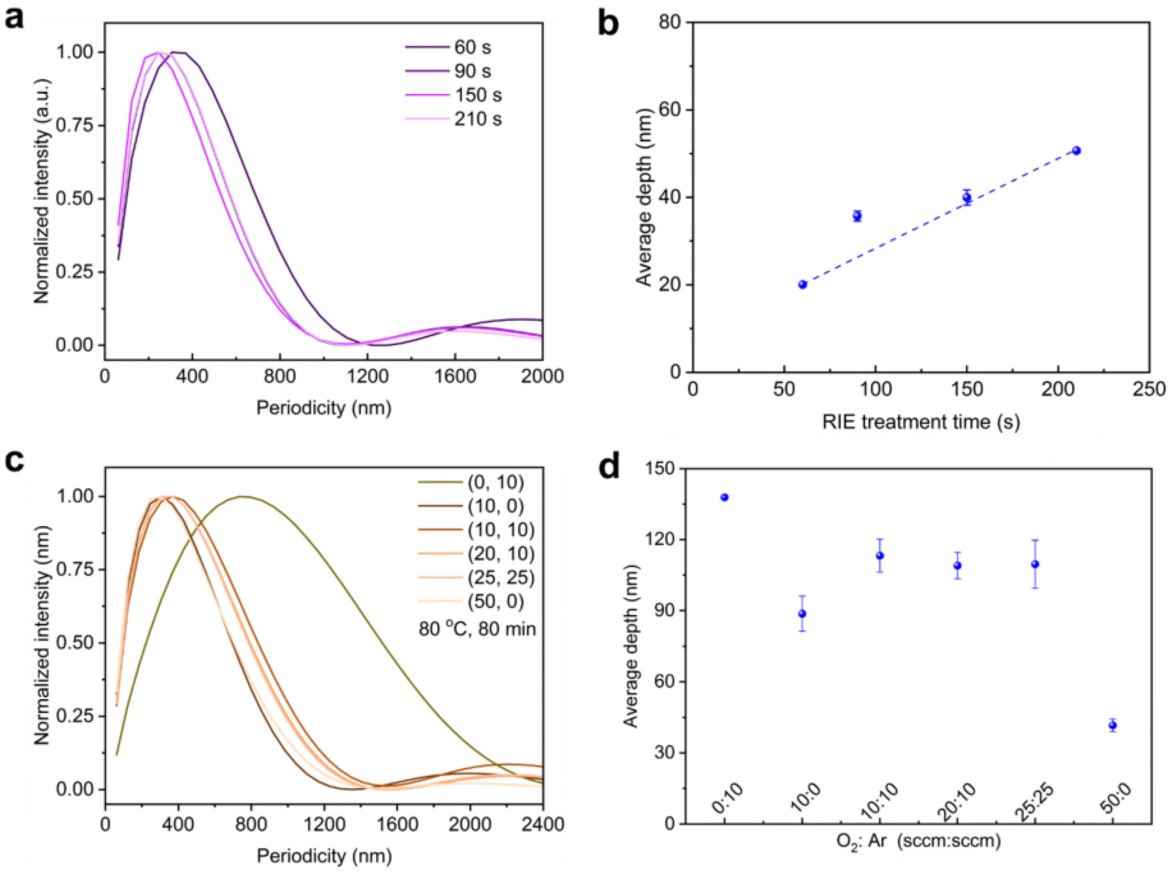

Supplementary Figure 4. Nanostructure control. The influence of the RIE treatment duration time (**a** and **b**) and the influence of the gas flow during RIE treatment (**c** and **d**), on the periodicity distribution **a, c** and average depth **b, d**. For **a** and **b**, the PDMS is consisted of a weight ratio of the base to curing agent of PDMS at 10:1 and cured at 80 °C for 80 min. RIE recipe: 20 W, 50 sccm O_2 for varied duration time. For **c** and **d**, PDMS is pretreated at 80 °C for 80 min, with a weight ratio of the base to curing agent at 10:1. RIE recipe: 50 W, 60 s, with various gas flow rates.

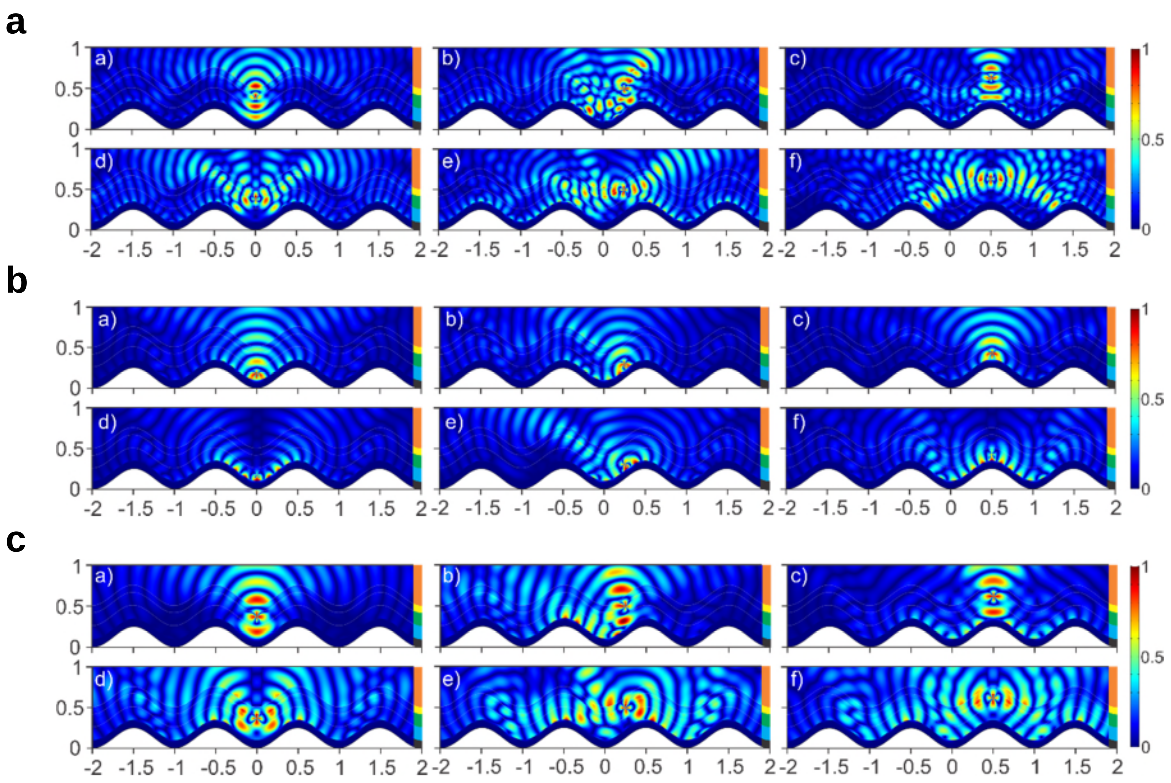

Supplementary Figure 5. Simulated optical filed distribution. A normalized steady state electric field for horizontal and vertical dipoles at wavelengths 430 nm (**a**), 510 nm (**b**) and 610 nm (**c**). For each wavelength, three different dipole positions at the bottom (first column), middle (second column) and on the top (third column) of sine texture (P = 1000 nm, h = 250 nm) are presented.

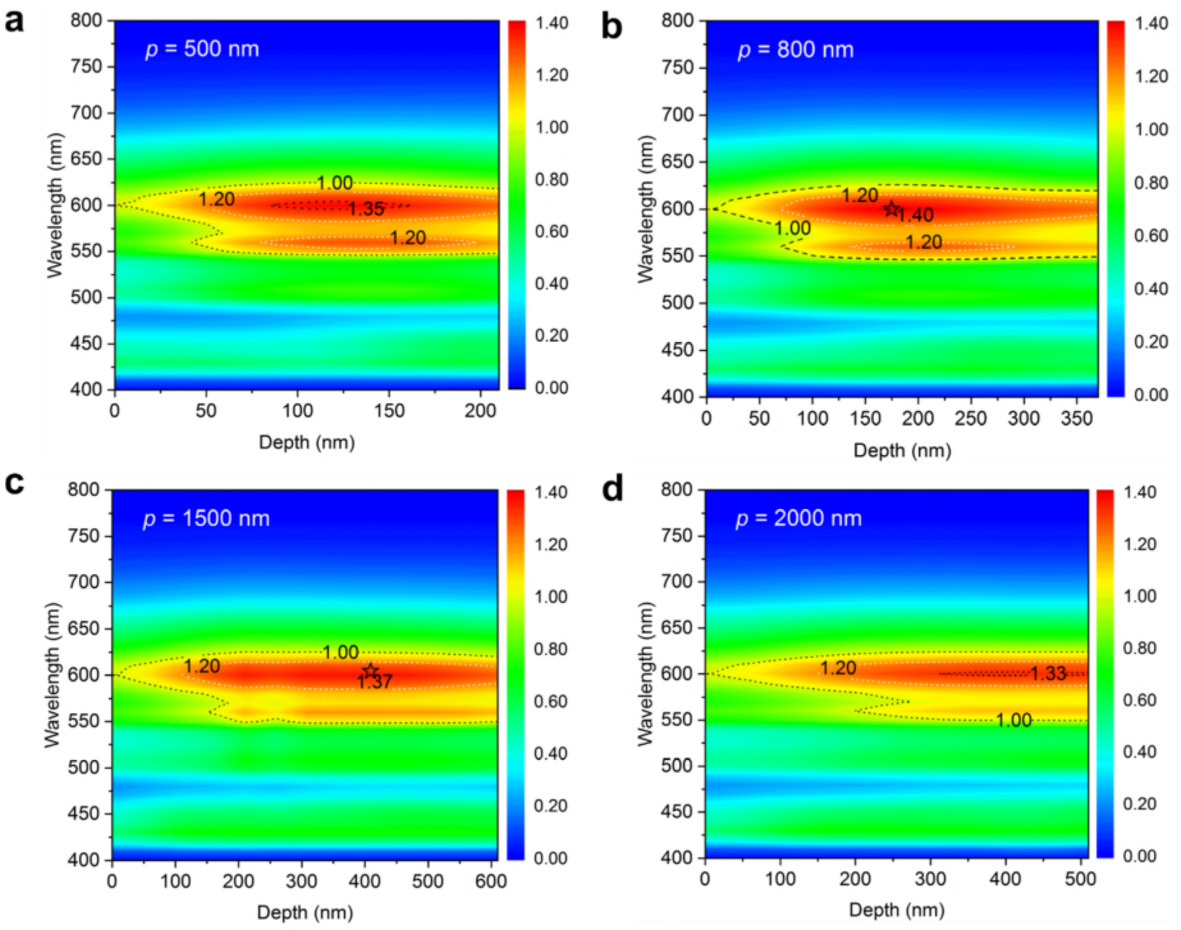

Supplementary Figure 6. Simulated wavelength dependent enhancement. White devices are based on nanostructures with different periodicity *p* by optical modelling. The depth of 0 represents the planar device. **a**, *p* = 500 nm; **b**, *p* = 800 nm; **c**, *p* = 1500 nm; **d**, *p* = 2000 nm.

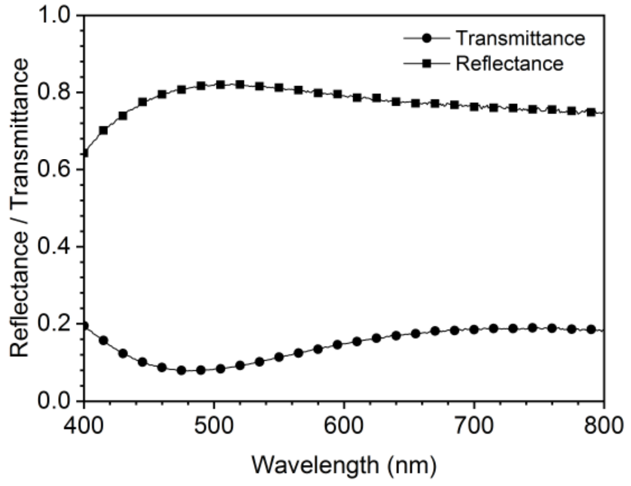

Supplementary Figure 7. The transmission and reflectance of the sputtered ITO. Thickness: 90 nm.

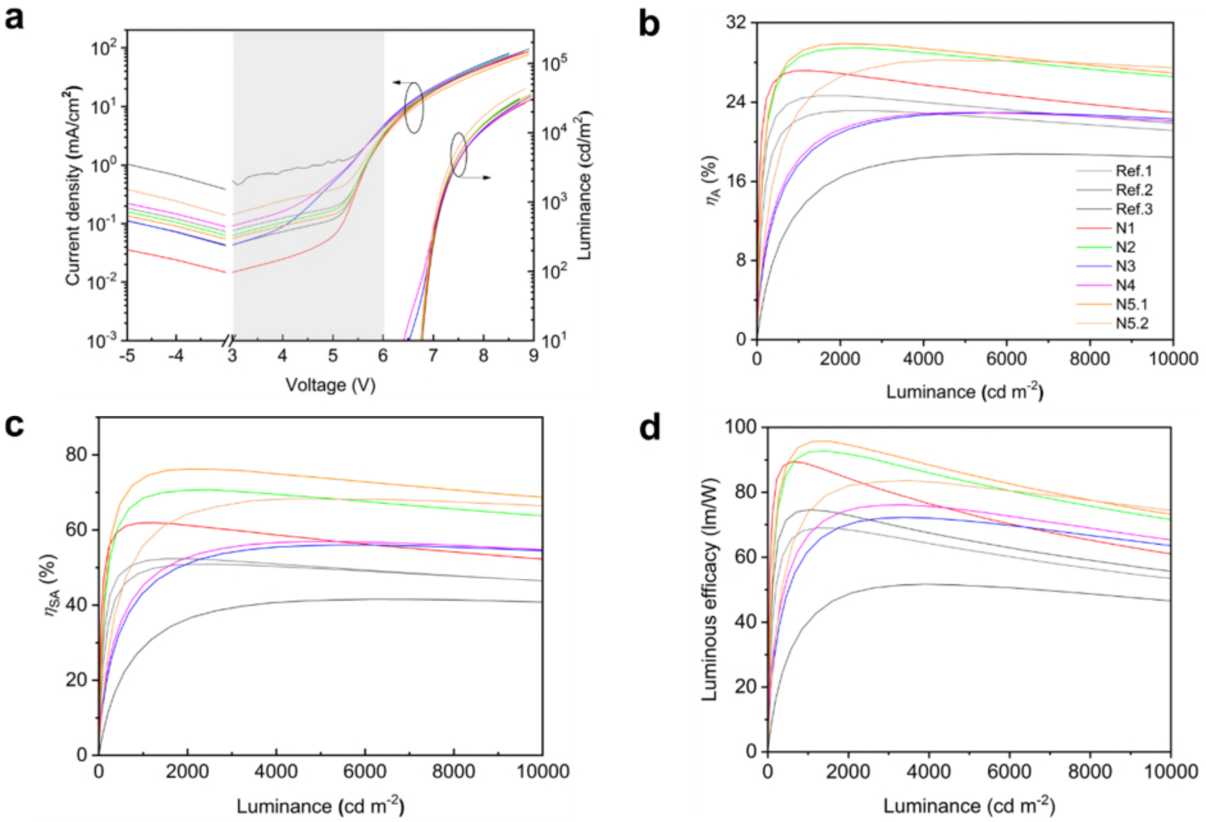

Supplementary Figure 8. OLEDs performance. **a**, the current density – voltage – luminance characteristics of investigated devices. The gray area indicates the pronounced influence of leakage current during that voltage range. **b**, luminance - η_A characteristics for all devices. **c**, luminance - η_A characteristics for all devices. **d**, luminance - luminous efficacy with a hemisphere lens.

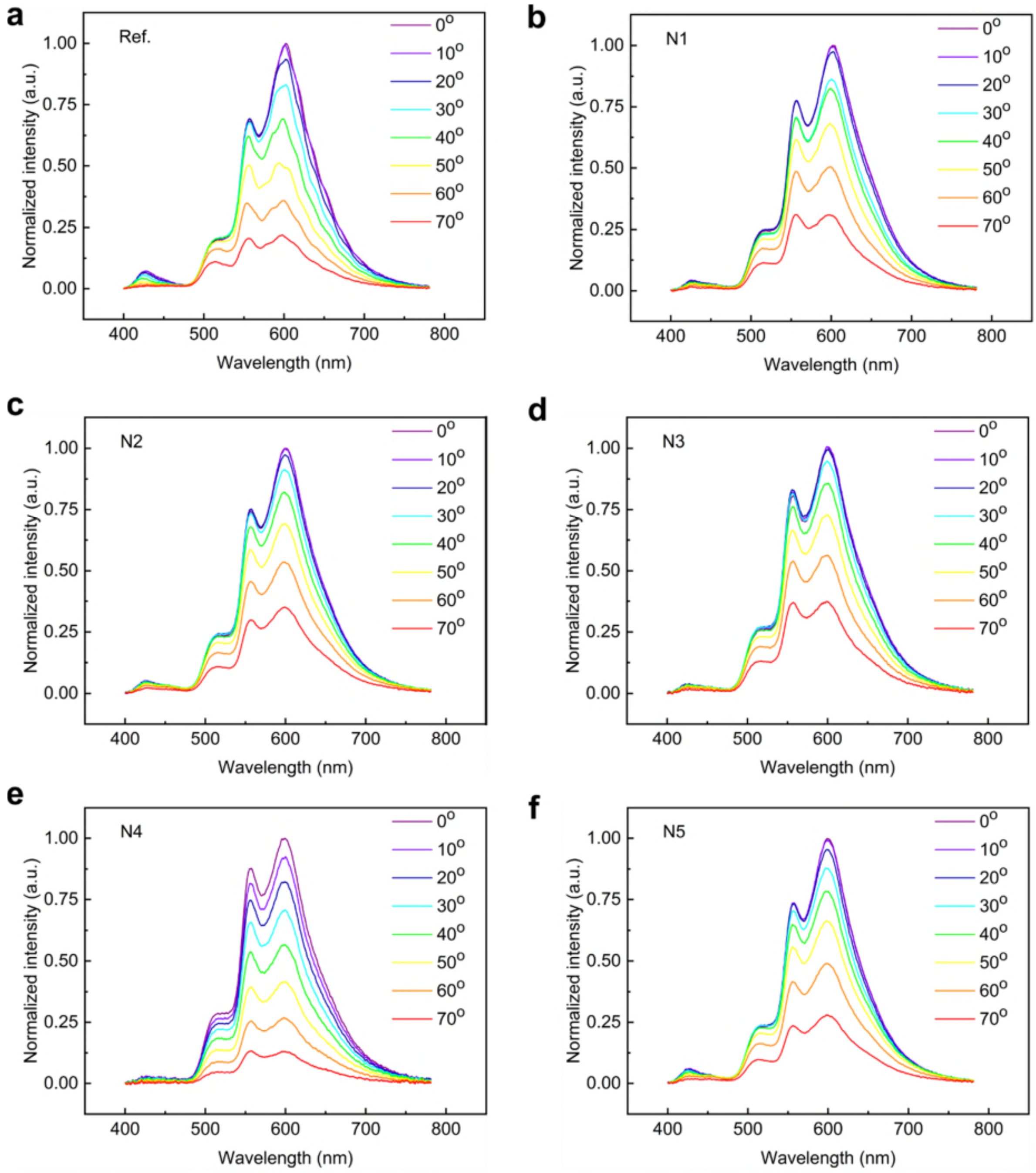

Supplementary Figure 9. OLEDs angular dependent EL spectra. **a**, Reference. **b**, N1. **c**, N2. **d**, N3. **e**, N4. **f**. N5.

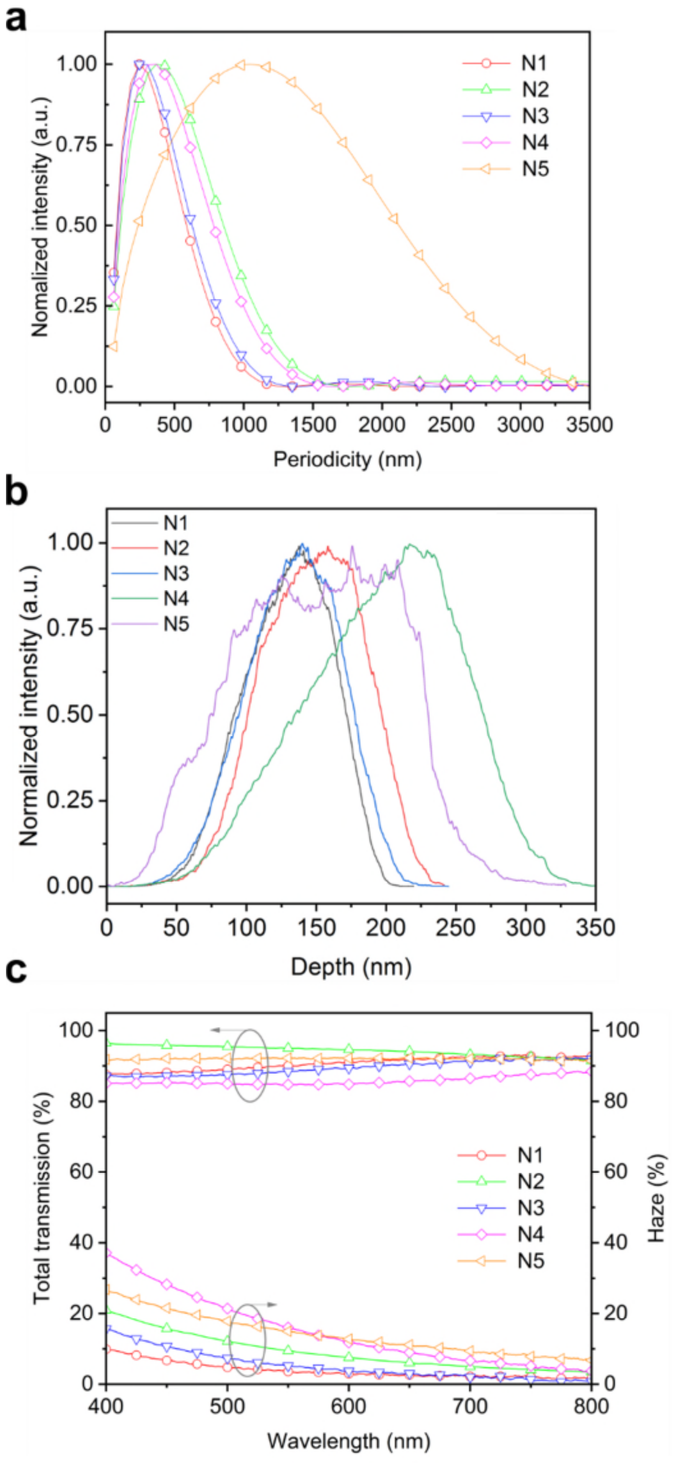

Supplementary Figure 10. Properties of the nanostructures used in OLEDs. **a**, the periodicity distribution. **b**, the depth distribution. **c**, the total transmission and haze factor.

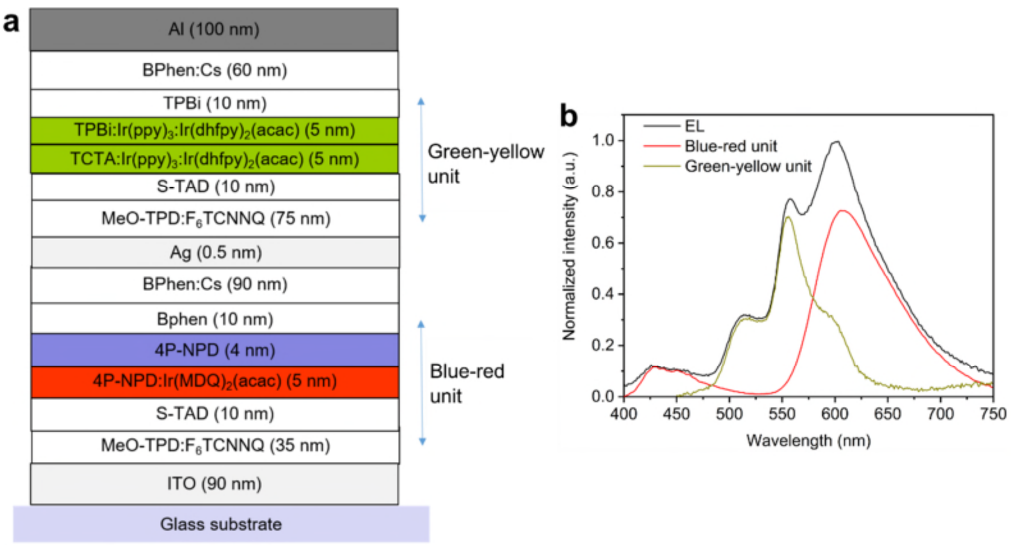

Supplementary Figure 11. The EL spectrum fitting for the planar white tandem OLED. **a**, OLEDs with two units: green-yellow and blue-red unit. **b**, EL spectrum fitting for each unit.

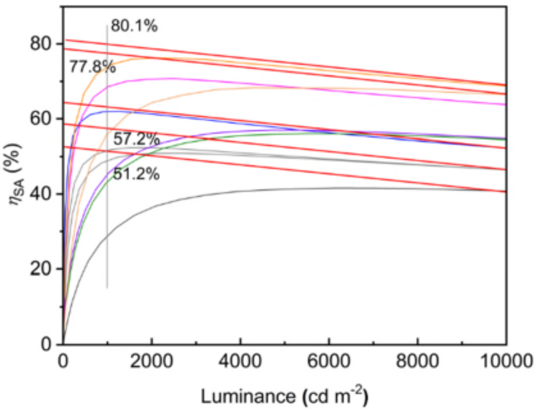

Supplementary Figure 12. Efficiency of light outcoupling structures. Recalculate the “real” maximum η_{SA} with the consideration of different leakage current.

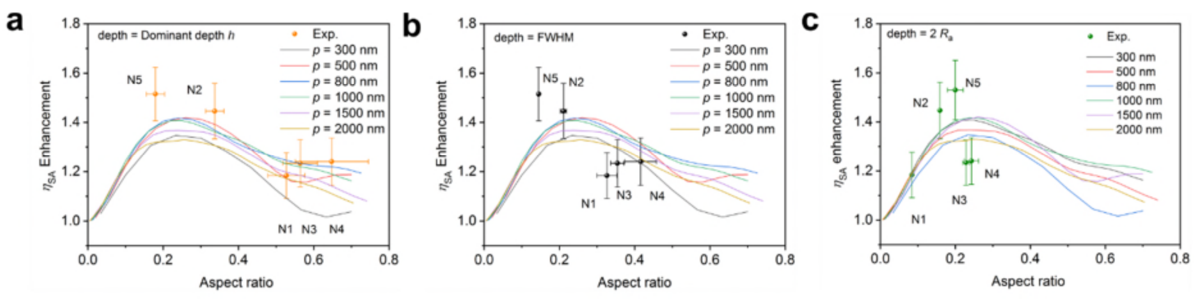

Supplementary Figure 13. Experimental aspect ratio. The different definition of depth (dots) for these nanostructures with simulated results (curves). **a**, depth = dominant depth; **b**, depth = FWHM; and **c**, depth = $2R_a$. The error bar is the standard deviation of at least 3 AFM measurements with high quality at different positions.

2. Supplementary Tables

Supplementary Table 1. Nanostructure uniformity: AR based on FWHM. The depth is based on the FWHM of the depth distribution determined from multiple AFM measurements at different sample positions. The dominant periodicity is obtained from AFM measurements with a resolution of 1024×1024. The proposed aspect ratio is calculated as FWHM / dominant periodicity.

Sample	FWHM of depth distribution (nm)						Dominant periodicity (nm)	Mean of AR	Standard deviation
	256×256			1024×1024					
N1	73.6	73.7	90.3	81	77.8	84.1	245.5	0.326	0.026
N2	90.9	91.6	-	99.9	96.1	95.6	449.9	0.211	0.008
N3	83.7	89	-	92	82.9	-	245.5	0.354	0.018
N4	128.1	141.5	-	159.9	132	-	337.5	0.416	0.042
N5	149	149	-	155.8	150.8	-	1043	0.145	0.003

Supplementary Table 2. Nanostructure uniformity: AR based on dominant depth. The depth is based on the dominant depth of the depth distribution determined from multiple AFM measurements at different sample positions. The dominant periodicity is obtained from AFM measurements with a resolution of 1024×1024. The proposed aspect ratio is calculated as dominant depth/dominant periodicity.

Sample	Dominant depth of depth distribution (nm)						Dominant periodicity (nm)	Mean of AR	Standard deviation
	256×256			1024×1024					
N1	123.4	110	142.9	137.8	126.2	136.1	245.5	0.527	0.049
N2	148.4	155.5	-	161.7	134	158.3	449.9	0.337	0.024
N3	141	122.8	-	149.8	140.3	-	245.5	0.564	0.046
N4	184.9	209.4	-	263.4	216.5	-	337.5	0.648	0.097
N5	213.5	157.5	-	202	175	-	1043	0.179	0.024

Supplementary Table 3. The atomic concentration of PDMS before and after RIE treatment.

	C	O	Si	O : Si ratio
As-prepared	46.2%	27.8%	26.0%	1.07
RIE treated	28.0%	46.4%	25.6%	1.81

Supplementary Table 4. The RIE treated PDMS with identical XPS spectra.

Pretreatment condition	O_2 (sccm)	Ar (sccm)	Power (W)	RIE duration time (s)
80 °C, 80 min	10	0	50	60
80 °C, 80 min	10	10	50	60
80 °C, 80 min	20	10	50	60
80 °C, 80 min	50	10	150	60

Supplementary Table 5. The ratio of η_{SA} / η_{A} for white OLEDs.

	Planar ref.	N1	N2	N3	N4	N5
η_{SA} / η_{A} *	2.18	2.29	2.41	2.45	2.48	2.47

* Calculated at 10,000 cd m^{-2}.

Supplementary Table 6. Comparison of internal outcoupling structures for white OLEDs. Improvements are calculated based on published values of external quantum efficiency (EQE) of white OLEDs without any outcoupling strategy (w/o), with only internal outcoupling strategy (w/in), with only external outcoupling strategy (w/ex), and with both internal and external outcoupling strategy (w/w). The enhancement factor is calculated in three different cases I: EQE (w/w) / EQE (w/o), II: EQE (w/in) / EQE (w/o), III: EQE (w/w) / EQE (w/ex) in this summary, depending on the data availability.

Light extraction strategies		External quantum efficiency (%)				Enhancement factor	Ref.
Internal	External	w/o	w/in	w/ex	w/w		
Low index grid[a,*]	Micro lens array	14.7	19	25	34	2.31[I], 1.29[II], 1.42[III]	[1]
Deterministic aperiodic nanostructures (DANs)[a,*]	-	26	56	-	-	2.15[II]	[2]
Vacuum nanohole array (VaNHA)[b,*]	Half-sphere lens	19.3	43.9	36.9	75.9	3.93[I], 2.27[II], 2.06[III]	[3]
High index substrate[a,*]	Index-matched half-sphere lens	13.1	14.4	24	34	2.60[I], 1.10[II], 1.42[III]	[4]
Nano-particle based scattering layers (NPSLs)[b,†]	Half-sphere lens	22	33	-	46	2.09[I], 1.5[II]	[5]
Subelectrode micro lens array (SEMLA)[a,*]	Micro lens array	16	20		27	1.69[I], 1.25[II]	[6]
Multifunctional Nanofunnel Arrays (NFAs)[b,*]	NFAs	12.7	20	19.6	29.4	2.31[I], 1.57[II]	[7]
Metal oxide nanostructures[c,†]	Half-sphere lens	14.3	20.3	26.6	35.5	2.48[I],1.42[II], 1.33[III]	[8]
RIE-induced nanostructures[c,†]	Half-sphere lens	20.4	27.3	44.4	69.0	3.38[I],1.34[II], 1.55[III]	This work

a, averaged peak value, if available. b, at 1,000 cd m^{-2}. c, average value at 10,000 cd m^{-2}.
*, single-unit white OLEDs. †, double-unit tandem white OLEDs.

Supplementary Table 7. Parameters for optical analysis of the planar white OLED.

	Emitter	η_{rad}	a	γ
Blue-red unit	4P-NPD	0.92[10]	0.33	0.73[11]
	Ir(MDQ)$_2$(acac)[12]	0.7	0.24	1
Green-yellow unit	Ir(ppy)$_3$[13]	0.76	0.31[14]	0.9
	Ir(dhfpy)$_2$ (acac)[15]	0.7	0.25[14]	1

Supplementary Table 8. The fraction of power for each unit of the planar white OLED.

	χ_{air} (%)	$\chi_{\mathrm{sub.}}$ (%)	$\chi_{\mathrm{wav.}}$ (%)	χ_{spp} (%)	Absorption (%)	Non-radiative loss (%)
Blue-red unit	9.8	7.0	27.4	11.9	11.2	32.7
Green-yellow unit	10.2	12.0	10.0	15.6	8.8	43.4

3. Supplementary Notes

Supplementary Note 1. Nanostructure fabrication

As discussed in the main text in the results part (section: nanostructure generation and characterization), the geometry of the nanostructures is randomly orientated on the surface of PDMS after the RIE-treatment. Statistically, the dimensional parameters including the periodicity and depth is quasi-periodic with a distribution, which can be measured by AFM. The experimental repeatability is monitored by measuring the periodicity distribution and average depth for the nanostructure generated in different batches with the same recipe as a tracking sample.

As shown in Supplementary Figure 3, in multiple batches from different time, the deviation of the dominant periodicity and the average depth of the tracking sample is very small, in contrast to the intentional changes induced through controlled variations of the processing recipe (see main text, Figure 2). For tracking samples generated during multiple, consecutive runs, the periodicity peaks at about 350 nm. Only very small deviation of average depth can be noted, as shown in Supplementary Figure 3b. Based on these results, we conclude that the process is facile and controllable, with good experimental repeatability.

We have carried out investigations of the uniformity of the nanostructure generation on PDMS by analyzing the local structure with AFM at different positions (at least four) of a given sample. From this, we calculate the mean value and the standard deviation of the experimental aspect ratio, which we use for our discussion.

As shown in Supplementary Figure 2, the periodicity distribution of a specific structure (N1) is almost the same for all these measurements, where each periodicity distribution is obtained by AFM measurement with a resolution of 1024×1024 at different positions. The dominant periodicity for N1 is 245.5 nm. There is only a slight deviation of the average depth at different positions, shown in Supplementary Figure 2. This is originated from the intrinsic difference at different positions and the experimental deviation for each AFM measurement.

The statistical values, including the full width at half maximum (FWHM) and the dominant depth of the depth distribution of all nanostructures at different positions, are summarized in Supplementary Table 1 and Supplementary Table 2. Analyzing the mean value and the standard deviation, the deviation of the proposed aspect ratio (AR) for the nanostructures at different position is very small, indicating that the nanostructures are uniform at different positions across the entire surface.

As shown in Supplementary Figure 4, the increase of RIE treatment time from 60 to 210 s

changes the dominant periodicity only slightly, while the average depth increases linearly from 20 to 50 nm. These results indicate that the treatment energy on the PDMS by either increasing the power or the time of the RIE treatment influences the depth of the nanostructure without significantly changing the periodicity distribution.

It is worth noting that a slight variation of the heating time of the PDMS layer can significantly affect the periodicity distribution, as shown in Fig. 2c. The shorter the pretreatment time, the larger the dominant periodicity. The dominant periodicity shifts from 610 nm for PDMS cured for 40 min, to 490 nm for samples cured for 60 min and further drops to 180 nm for samples pretreated for 80 min or longer. In addition, samples thermally pretreated for 40 and 60 min give a broader periodicity distribution than these pretreated for 120 min or 160 min, with the main difference over the range of large periodicities. However, the average depth corresponds to 200 nm for PDMS cured for 40 min, which is about 6 times larger compared to the PDMS cured for 160 min (35 nm), as shown in Fig. 2d. These results demonstrate the possibility of manipulating the periodicity distribution and depth simultaneously.

Further investigations indicate that the weight ratio of the base to the curing agent of PDMS can also tune the periodicity distribution and the average depth. As depicted in Fig. 2e, the dominant periodicity increases from 120 to 250 nm when the weight ratio of the base to the curing agent raises from 5:1 to 20:1. Meanwhile, the average depth grows when the ratio is increasing, as shown in Fig. 2f. The average depth is 40 nm for the sample with a ratio of 20:1, which is about 3 times higher compared to the sample with a ratio of 5:1 (12 nm).

As shown in Supplementary Figure 4, when fixing the RIE treatment power and time, the change of gas species can influence the periodicity distribution and the average depth dramatically. In general, samples treated with mere argon give larger nanostructures compared to samples treated with O_2 only. The Ar flow with a rate of 10 sccm leads to nanostructures with a dominant periodicity of 730 nm and an average depth of 140 nm, while a 10 sccm O_2 flow can only induce a pattern with a dominant periodicity of 300 nm and an average depth of 90 nm. Varying the ratio of O_2 and Ar can slightly tune the periodicity distribution and average depth. The increase of oxygen flow in the mixed gas can slightly decrease the average depth of the nanostructure.

In summary, the various options to control the deformation of PDMS by RIE process conditions significantly increase the design freedom of photonic nanostructures for OLED light outcoupling compared to the lithography, molding or nanoimprinting techniques. Only this control makes it possible to tailor-make such structures for a given OLED architecture,

which is of key importance, as the latter can vary strongly depending on their application.

Supplementary Note 2. Optical analysis of loss channels for planar white OLEDs

The external quantum efficiency for a single unit OLED could be obtained by:

$$\eta_{\mathrm{ext}} = \xi\gamma \int_{\lambda} S_{\mathrm{el}}(\lambda) \frac{F(\lambda)\eta_{\mathrm{rad}}}{1-\eta_{\mathrm{rad}}+F(\lambda)\eta_{\mathrm{rad}}} \frac{U(\lambda)}{F(\lambda)} d\lambda \ \# \ (1)$$

where ξ is the proportion of the useful excitons, γ the electrical efficiency, the $S_{\mathrm{el}}(\lambda)$ the normalized luminescent spectrum of the emitter, $F(\lambda)$ the total radiated power (Purcell factor), η_{rad} the effective radiative quantum yield of the exciton, $U(\lambda)$ the outcoupled power at wavelength λ.

Since the planar white OLED is consisted of two units: one is fluorescent blue emission and phosphorescent red emission, the other is green-yellow emission unit with double emission layers. We here calculate the loss channels for each unit separately,[9] where the electroluminescent spectrum $S_{\mathrm{el}}(\lambda)$ for each unit is fitted from the emission spectrum obtained in the integrating sphere measurement, as shown in Supplementary Figure 11. To calculate the energy losses for each unit, the other unit is treated as functional organic layers without emission. The effective radiative quantum yield η_{rad}, the anisotropy factor a, the electrical efficiency γ for each emitter are obtained from literature, as summarized in the Supplementary Table 7.

As summarized in Supplementary Table 8, the majority of non-radiative losses is coming from a medium effective radiative efficiency and the electrical efficiency, giving the internal quantum efficiency for the planar tandem white OLED about 125%, which is very close with the previously reported tandem white OLEDs based on 4P-NPD system.[2,3]

Supplementary Note 3. Efficiency of light outcoupling structures (ELOS)

The ELOS, which can be defined as[16]:

$$\eta_{\mathrm{ELOS}} = \frac{\eta_{\mathrm{SA}}^{\mathrm{w/}} - \eta_{\mathrm{SA}}^{\mathrm{w/o}}}{\chi_{\mathrm{wav.}} + \chi_{\mathrm{SPP}}} \# (2)$$

where $\eta_{\mathrm{SA}}^{\mathrm{w/}}$ represents the maximum η_{SA} with nanostructures, $\eta_{\mathrm{SA}}^{\mathrm{w/o}}$ the maximum η_{SA} without nanostructures.

To get the "real" η_{SA} maxima without the influence of leakage current, we here choose the device with the minimum leakage current as a reference and assume all these devices have the same roll-off, as shown in Supplementary Figure 12. The leakage current for all devices is in the range from 0.02 to 0.4 mA cm^{-2} under reverse voltage of 4 V. Compared to our previous reports about devices with the same stack on the commercial ITO, the leakage current is about 1-2 orders of magnitude higher. It is most likely both the perturbation of the nanostructures and the intrinsically higher roughness of ITO films without annealing process contribute to the higher leakage current. The deficiency of the ITO anode should be one of the main sources leading to the high leakage current. As presented in the Supplementary Figure 8, the leakage current for the reference samples without the nanostructure is comparable to the structured device, demonstrating that the roughness of the flat ITO anode could already give rise to high leakage current. The presence of photoresist NOA 63 under the anode can only tolerate a thermal treatment with highest temperature about 60°C (data from the supplier).[17] As demonstrate by Ref. 48 in the main text, higher annealing temperature can reduce the roughness of ITO and enhance the conductivity.

The η_{ELOS} with different device combinations can be calculated. For example:

$$\eta_{\mathrm{ELOS,1}} = \frac{\eta_{\mathrm{SA}}^{\mathrm{w/}} - \eta_{\mathrm{SA}}^{\mathrm{w/o}}}{\chi_{\mathrm{wav.}} + \chi_{\mathrm{SPP}}} = \frac{80.1\% - 51.2\%}{27.4\% + 11.9\% + 10.0\% + 15.6\%} = \frac{28.9\%}{64.9\%} = 44.5\% \# (3)$$

Averaging all the combinations, given the averaged $\eta_{\mathrm{ELOS,\ ave}}$ as 36.6% for devices with N5.

Supplementary Note 4. Experimental aspect ratio

As shown in Supplementary Figure 1 and Supplementary Figure 10, the depth of these quasi-periodic nanostructures is widely distributed from 0 nm to more than 300 nm. The average roughness R_a, which has been widely used for describing the depth of nanostructures,[18] is mathematically defined as:[19]

$$R_a = \frac{1}{N}\sum_{1}^{N}\left(h_n - \bar{h}\right) \#(4)$$

Where N represents the total data points of AFM measurements, h_n is the height for a specific point n and $\bar{h}$ is the average height for all positions.

For the nanostructure generation mechanism investigated in this work, it is more reasonable to treat the average depth as $2R_a$, since the generation of nanostructures is in response to the compressive stress release among the entire PDMS surface, as discussed in the main text, while the average roughness is taking all the position into account.

However, for outcoupling trapped photons in OLEDs, it is that the real geometry of the nanostructures makes the optical influence, not the mathematically averaged depth. Therefore, a definition of the depth close to the real geometry would be better for outcoupling purposes.

The area normalized density function of the depth distribution in the AFM measurement is defined as:[19]

$$\int_{-\infty}^{\infty}\rho(h)dh = 1 \# (5)$$

where the $\rho(h)$ is the density function for the depth among all the depth. The dominant depth of the density function $\rho(h)$ represents the depth with the highest density among all the depth measured. In this point of view, the dominant depth can represent the optical effects from the real nanostructures.

It should be noted that the experimental error can influence the absolute value of the dominant depth h among a specific depth distribution, resulting from the shift of the lowest depth (0 nm) in AFM measurements. Averaging dominant depth among different high-resolution AFM measurements at a low noise level can statistically reduce the error.

For periodic 2D grating or pillar structures, the relative difference of the peaks of the depth density function $\rho(h)$ can represent the real depth for these structures.[16] For complicated nanostructures with a broad depth distribution in this work, there are hardly two peaks in the density function of depth distribution, shown in Supplementary Figure 1 and Supplementary Figure 8. A compromised way is using the full width at half maximum (FWHM) of the

density function. However, there is a lack of clear physical meaning for the FWHM for these nanostructures.

It is worth noting that when treating the dominant depth *h* among the depth distribution as the depth of the nanostructure, a good agreement of the experimental and simulation results can be found, as shown in Supplementary Figure 13. For the other two cases, as discussed previously, because of the weakness of physical meaning for the FWHM and the improper description for the outcoupling effect with $2R_a$, they both give larger deviation from the simulation results. However, for such a nanostructure system bridging chaos and order, the evaluation of the depth by the dominant depth *h* among the density function can also bring some system errors, e.g. the determination of the 'zero' depth cannot be given with absolute certainty. Still, the similar trend and close value to the simulation results gives a hint that this could be a useful and, yet very simple and first estimation to understand such a complicated system.

Supplementary References

1. Sun, Y. & Forrest, S. R. Enhanced light out-coupling of organic light-emitting devices using embedded low-index grids. *Nat Phot.* **2,** 483–487 (2008).
2. Ou, Q.-D. *et al.* Extremely Efficient White Organic Light-Emitting Diodes for General Lighting. *Adv. Funct. Mater.* **24,** 7249–7256 (2014).
3. Jeon, S. *et al.* High-Quality White OLEDs with Comparable Efficiencies to LEDs. *Adv. Opt. Mater.* **17013491,** 1–8 (2018).
4. Reineke, S. *et al.* White organic light-emitting diodes with fluorescent tube efficiency. *Nature* **459,** 234–238 (2009).
5. Chang, H. W. *et al.* Nano-particle based scattering layers for optical efficiency enhancement of organic light-emitting diodes and organic solar cells. *J. Appl. Phys.* **113,** (2013).
6. Qu, Y., Kim, J., Coburn, C. & Forrest, S. R. Efficient, Non-Intrusive Outcoupling in Organic Light Emitting Devices Using Embedded Microlens Arrays. *ACS Photonics* **5,** 2453–2458 (2018).
7. Zhou, L. *et al.* Efficiently Releasing the Trapped Energy Flow in White Organic Light-Emitting Diodes with Multifunctional Nanofunnel Arrays. **25,** 2660–2668 (2015).
8. Kim, Y. H. *et al.* We Want Our Photons Back: Simple Nanostructures for White Organic Light-Emitting Diode Outcoupling. *Adv. Funct. Mater.* **24,** 2553–2559 (2014).
9. Furno, M., Rosenow, T. C., Gather, M. C., Lüssem, B. & Leo, K. Analysis of the external and internal quantum efficiency of multi-emitter, white organic light emitting diodes. *Appl. Phys. Lett.* **101,** (2012).
10. Schwartz, G., Pfeiffer, M., Reineke, S., Walzer, K. & Leo, K. Harvesting triplet excitons from fluorescent blue emitters in white organic light-emitting diodes. *Adv. Mater.* **19,** 3672–3676 (2007).
11. Rosenow, T. C. *et al.* Highly efficient white organic light-emitting diodes based on fluorescent blue emitters. *J. Appl. Phys.* **108,** (2010).
12. Schmidt, T. D. *et al.* Evidence for non-isotropic emitter orientation in a red phosphorescent organic light-emitting diode and its implications for determining the emitter's radiative quantum efficiency. *Appl. Phys. Lett.* **99,** 2012–2015 (2011).
13. Furno, M., Meerheim, R., Hofmann, S., Lüssem, B. & Leo, K. Efficiency and rate of spontaneous emission in organic electroluminescent devices. *Phys. Rev. B* **85,** 1–21 (2012).
14. Graf, A. *et al.* Correlating the transition dipole moment orientation of phosphorescent

emitter molecules in OLEDs with basic material properties. *J. Mater. Chem. C* **2,** 10298–10304 (2014).

15. Hofmann, S., Furno, M., Lüssem, B., Leo, K. & Gather, M. C. Investigation of triplet harvesting and outcoupling efficiency in highly efficient two-color hybrid white organic light-emitting diodes. *Phys. Status Solidi Appl. Mater. Sci.* **210,** 1467–1475 (2013).
16. Will, P. *et al.* Efficiency of Light Outcoupling Structures in Organic Light Emitting Diodes: 2D TiO_2 Array as a Model System. *Adv. Funct. Mater.* 1901748 (2019).
17. NOA63. Available at: https://www.norlandprod.com/adhesives/NOA 63.html. (Accessed: 6th April 2019)
18. Lee, I. *et al.* Spontaneously Formed Nanopatterns on Polymer Films for Flexible Organic Light-Emitting Diodes. *Small* **11,** 4480–4484 (2015).
19. Statistical Analysis. Available at: http://gwyddion.net/documentation/user-guide-en/statistical-analysis.html. (Accessed: 4th February 2019)
